# 肺癌侧群细胞microRNA表达谱检测及初步分析

**DOI:** 10.3779/j.issn.1009-3419.2010.07.02

**Published:** 2010-07-20

**Authors:** 小涛 徐, 晓 陆, 婧 孙, 永前 束

**Affiliations:** 210029 南京，南京医科大学第一附属医院肿瘤内科 Cancer Biotherapy Center, the First Affiliated Hospital of Nanjing Medical University, Nanjing 210029, China

**Keywords:** 肿瘤干细胞, 侧群细胞, microRNA, 基因芯片, Cancer stem cell, Side population cell, microRNA, Microarray

## Abstract

**背景与目的:**

目前研究认为侧群细胞（side population cell, SP cell）富集了肿瘤干细胞，是肿瘤生长和发展的根源；并且SP细胞对放化疗高度耐受，成为肿瘤复发转移的重要因素。为此，我们探讨了肺癌SP细胞与非SP细胞（non-SP）miRNA分子表达谱差异，旨在为进一步研究miRNA在肺癌干细胞中的作用机制打下基础。

**方法:**

利用Hoechst 33342染料法，通过流式细胞仪紫外激光分选功能分选出A549细胞中的SP细胞和non-SP细胞，分别采用Trizol一步法提取两者的总RNA，利用miRNA芯片检测系统进行miRNA表达谱差异分析。

**结果:**

肺癌SP细胞和non-SP细胞miRNA表达谱存在明显差异。与non-SP细胞相比，在SP细胞中上调2倍以上的miRNA有9条，下调2倍以上的miRNA有25条。

**结论:**

差异表达的miRNA可能参与肺癌干细胞的发生发展过程，为进一步揭示肺癌干细胞发生的分子机制提供了依据。

## Abstract (en)

**Background and objective:**

Recent studies indicate that the side population (SP) which is an enriched source of cancer stem cells (CSCs) is the root cause of tumor growth and development. SP appears to be highly resistant to chemo- and radio-therapy which becomes an important factor in tumor recurrence and metastasis. The aim of this study is to determine the difference of microRNA expression profiles between SP cells and non-SP cells so as to lay necessary basis for research on the function of miRNA in lung cancer stem cells.

**Methods:**

SP and non-SP cells were isolated using flow cytometry and Hoechst 33342 dye efflux assay from human lung adenocarcinoma A549 cell. The total RNA was extracted. The microarray detection system was employed to analyze whether there was difference in miRNA expression profile between SP and non-SP cells.

**Results:**

A total of 85 differentially expressed miRNA were found, including 32 over-expression and 53 low-expression miRNA in SP.

**Conclusion:**

miRNA may play important roles in tumorigenesis of lung cancer stem cell. The study of miRNA contributes to elucidate the molecular mechanism of lung cancer stem cell.

肿瘤干细胞是肿瘤细胞群体中一小部分具有自我更新和多向分化能力的起始细胞，这部分细胞对放化疗耐受，转移潜力高，也是肿瘤异质性形成的主要原因^[1]^。侧群细胞（side population cell, SP cell）是1996年/祖细胞分离时发现的一群特殊细胞。现发现SP细胞广泛分布于多种成体组织、胚胎和多种肿瘤细胞中，具有类似干细胞的自我更新能力和多向分化潜能。由于肿瘤干细胞表面高表达ABCG2转运体，能够高效外排荧光染料Hoechst 33342，可以通过流式细胞仪来分选。Ho等^[3]^研究证明肺癌SP细胞具有更高的致瘤性和化疗抵抗性，具有肿瘤干细胞特性，是肿瘤发生维持、转移、复发的重要因素。

miRNA是一类内源性单链小片段非编码RNA分子，Goodell等^[2]^利用Hoechst染料通过流式细胞术进行造血干在基因表达调控中起着非常重要的作用^[4]^。目前已发现多种miRNA在肿瘤中起着癌基因和抑癌基因的作用，对肿瘤细胞分化和凋亡等过程具有重要的调节功能^[5]^；对肿瘤干细胞相关miRNA的研究发现，miRNA通过多重调控靶基因来调节肿瘤干细胞的自我更新和多向分化^[6]^。

基于目前对肿瘤干细胞的研究结果，我们推测在肺癌干细胞中可能存在某些特异miRNA对调节肿瘤干细胞的生命活动起着重要作用。因此我们利用高通量基因芯片技术，检测肺癌A549细胞株中SP和non-SP的miRNA表达谱，筛选出差异miRNA，为进一步研究这些miRNA在肺癌干细胞中的功能打下基础。

## 1 材料与方法

### 1.1 材料

人肺腺癌A549细胞系购于上海麦莎生物科技有限公司；RPMI-1640培养基、胎牛血清、胰酶为GIBCO公司产品；Hoechst 33342、verapamil、碘化丙啶购于美国Sigma公司；Trizol试剂为Invitrogen公司产品，miRNA芯片由美国LC Sciences公司提供。

### 1.2 方法

#### 1.2.1 细胞培养

A549细胞用含10%胎牛血清、100 U/mL青霉素和100 U/mL链霉素的DMEM培养液，在37 ℃、5%CO_2_饱和湿度条件下培养；0.02%EDTA和0.25%胰蛋白酶的1:1混合液消化、传代。实验采用对数生长期细胞。

#### 1.2.2 细胞分选

利用BD FACS VantageSE流式细胞仪分选A549细胞系SP及non-SP细胞。收集对数生长期细胞，制备成单细胞悬液，1 200 r/min离心5 min，PBS洗涤2次，用预热37 ℃的含10%胎牛血清的Hanks’盐平衡液重悬，调整细胞数至1×10^6^/mL。一组加入Hoechst 33342至终浓度为5 mg/L，另一组同时加入verapamil至终浓度为50 μmol/L，37 ℃水浴90 min，每隔15 min摇匀一次，孵育结束后，1 200 r/min离心5 min，弃上清，用预冷的含2%小牛血清的PBS洗1次，重悬于含10%胎牛血清的Hanks’盐平衡液中，加入PI至终浓度为2 mg/L，上流式细胞仪检测、分选细胞。Hoechst 33342的激发光为352 nm紫外光，424/44带通收集蓝光，630/22带通收集红光。P1的激发光为488 nm蓝光，用575/26带通收集红光。

#### 1.2.3 总RNA提取及miRNA芯片分析

Trizol法提取组织中的总RNA，通过异丙醇沉淀法浓缩RNA，用分光光度计定量，甲醛变性胶电泳质检总RNA质量。所用芯片由美国LC Sciences公司制作。通过YM-100（Milllpore）微离心过滤柱得到片段 < 300 nt的小RNA。Poly（A）聚合酶在分离到的小RNA 3’端加上Poly（A）尾，再将一个寡聚核苷酸标记与该Poly（A）尾巴连接用于后续的荧光标记。双样品实验中，用两个不同的标记物来标记两个RNA样品。杂交反应通过微循环泵杂交仪器在µParaflo^TM^微流体芯片上过夜，杂交使用含有25%的甲酰胺的100 μL 6×SSPE缓冲液，杂交温度34 ℃，杂交检测使用Cy3和Cy5特异性荧光标记。

### 1.3 数据分析

采用激光扫描仪采集杂交图像，Array-Pro软件对杂交图像进行数字化转换。数据处理和分析首先扣除背景，计算重复点平均值和标准偏差，然后通过LOWESS过滤进行标准化。本实验为双色标记实验，计算两种检测信号的比值（log2）和*t*检验的*P*值，以*P* < 0.01为差异有统计学意义。

## 2 结果

### 2.1 A549细胞SP检测及分选结果

运用Hoechst染色法通过流式细胞仪检测到A549细胞株中存在约1.1%的SP细胞，该部分侧群细胞在加入ABCG2通道抑制剂verapamil后比例下降至0.2%（图 1）。

**1:**
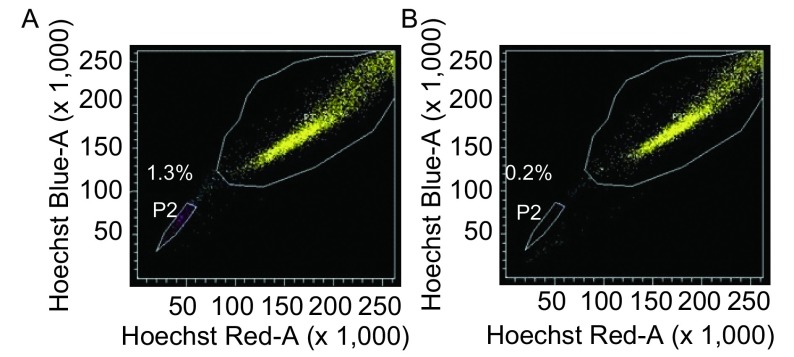
A549经verapamil处理后SP比例由1.1%（A）降为0.2%（B） A549 cells contained 1.1% of SP cells (A), dropped to 0.2% after treatment with the selective ABC transporter inhibitor verapamil (B)

### 2.2 总RNA提取结果

Trizol一步法提取SP与non-SP细胞总RNA，通过异丙醇沉淀法浓缩RNA，用分光光度计定量。根据RNA在*A*_260_/*A*_280_值1.80-2.10之间以及甲醛变性凝胶电泳28S、18S RNA条带比值≥1.5，鉴定RNA纯度及其完整性（图 2）。

**2:**
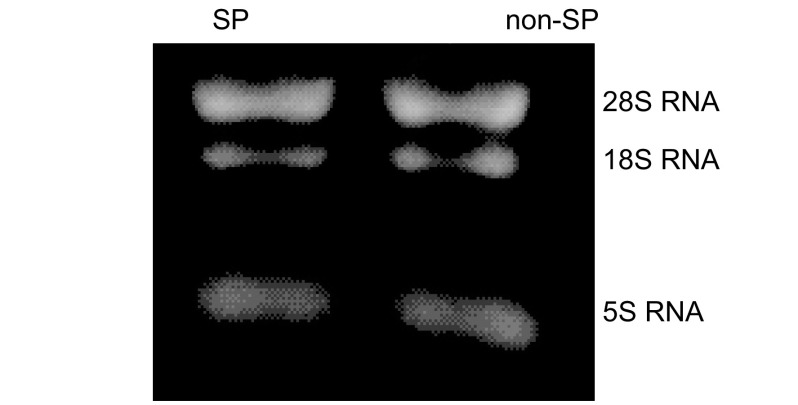
SP与non-SP总RNA凝胶电泳 Total RNA isolated from SP cells and non-SP cells by gel electrophoresis

### 2.3 miRNA芯片数据分析结果

芯片结果见图 3所示，其中，A：non-SP-Cy3信号强度图，B：SP-Cy5信号强度图，C：Cy3/Cy5信号比值图。当Cy3信号高于Cy5信号时，色彩显示为绿色；当Cy3与Cy5信号相当时，色彩显示为黄色；当Cy5信号高于Cy3信号时，色彩显示为红色（图 3）。结果显示，在SP与non-SP细胞中，miRNA表达存在显著差异。与non-SP相比，在SP细胞中上调2倍以上的miRNA有9条，下调2倍以上的miRNA有25条（表 1，表 2）。

**3:**
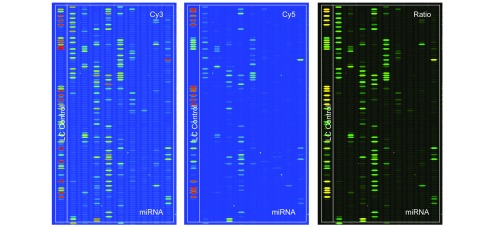
SP/non-SP miRNA芯片图谱。图像以伪色显示扩大可视的动态范围。在Cy3和Cy5的信号强度图像中，随着信号强度从1增加到65 535，对应的色彩会从蓝-绿-黄-红进行渐进变化。在Cy3/Cy5信号比值图中，当Cy3信号高于Cy5信号时，色彩显示为绿色；当Cy3信号与Cy5信号相当时，色彩显示为黄色；当Cy5信号高于Cy3信号时，色彩显示为红色。 SP/non-SP miRNA chips images. The images are displayed in pseudo colors so as to expand the visual dynamic range. In the Cy3 and Cy5 intensity images, as the signal intensity increases from 1 to 65 535 the corresponding color changes from blue to green, then to yellow, and finally to red. In the Cy3/Cy5 ratio image, when Cy3 level is higher than Cy5 level the color is green; when Cy3 level is equal to Cy5 level the color is yellow; and when Cy5 level is higher than Cy3 level the color is red.

**1:** 上调2倍以上的miRNAs Over-expression miRNAs (fold changes≥2)

No.	miRNA	non-SP signal	SP signal	log2 (SP/non-SP)
1	hsa-miR-548m	49.93	592.08	3.42
2	hsa-miR-498	76.06	599.62	2.98
3	hsa-miR-1281	51.24	283.11	2.63
4	hsa-miR-940	83.53	421.24	2.27
5	hsa-miR-1274b	715.29	2 692.88	1.87
6	hsa-miR-1274a	677.10	2 442.08	1.85
7	hsa-miR-1974	10 831.77	32 964.3	1.62
8	hsa-miR-210	506.42	1 426.69	1.48
9	hsa-miR-1975	8 589.10	18 217.90	1.10

**2:** 下调2倍以上的miRNAs Low-expression miRNAs (fold changes≥2)

No.	miRNA	non-SP signal	SP signal	log2 (SP/non-SP)
1	hsa-miR-98	767.58	83.77	-3.29
2	hsa-miR-31	613.77	73.05	-3.07
3	hsa-miR-16-2^*^	278.24	39.69	-2.92
4	hsa-miR-15b^*^	438.11	59.69	-2.88
5	hsa-let-7e	2 669.92	407.08	-2.71
6	hsa-miR-30e^*^	238.74	39.98	-2.70
7	hsa-let-7c	4 255.33	802.33	-2.68
8	hsa-miR-125a-5p	515.43	127.42	-2.33
9	hsa-miR-20b	2 918.43	822.01	-2.04
10	hsa-miR-29c	495.35	150.20	-1.74
11	hsa-miR-29b	1 676.73	538.58	-1.64
12	hsa-miR-7	353.07	116.15	-1.63
13	hsa-miR-365	340.85	110.91	-1.62
14	hsa-miR-148b	941.97	309.18	-1.60
15	hsa-let-7d	5 169.73	1 761.86	-1.55
16	hsa-miR-214	441.08	149.59	-1.55
17	hsa-miR-27a	4 049.62	1 424.87	-1.47
18	hsa-miR-181b	925.42	317.64	-1.47
19	hsa-miR-224	2 821.07	1 027.39	-1.46
20	hsa-let-7g	3 489.87	1 332.90	-1.34
21	hsa-miR-186	947.75	358.90	-1.33
22	hsa-miR-101	850.80	337.96	-1.33
23	hsa-miR-30a^*^	448.89	190.98	-1.27
24	hsa-miR-30e	2 357.83	991.42	-1.22
25	hsa-miR-27b	3 067.14	1 404.30	-1.10

## 3 讨论

肿瘤干细胞学说的提出为肿瘤研究提供了一个新的视角，目前在肿瘤干细胞研究方面已经取得了很多令人振奋的结果。运用肿瘤干细胞理论可以比较好地解释目前临床上所遇到的肿瘤放化疗耐受及肿瘤转移复发的困惑。为了进一步研究肿瘤干细胞与一般肿瘤细胞的差异，我们从miRNA——这一新发现的重要基因调控分子出发，利用高通量基因芯片技术检测两者之间的miRNA表达谱差异，希望找到肿瘤干细胞相关性miRNA。

Northern blot分析和克隆技术均可用于miRNAs表达分析，但二者都存在着工作量大、敏感性较低的缺点，无法用于高通量的miRNAs表达谱分析。而微阵列技术可以一次性同时检测数百个miRNA的表达。miRNA寡核苷酸芯片是目前研究肿瘤特异性miRNA表达的最常用的高通量方法。

本研究所使用基因芯片及其质控探针由美国LC Sciences（联川）公司提供，采用μParaflo^TM^ microRNA（miRHuman 13.0版）微阵列芯片技术平台，涵盖了Sanger miR-Base序列数据库中所有物种。每张芯片上的检测探针在杂交反应中均进行了至少3个重复。

根据芯片结果我们发现，肺癌SP细胞和non-SP细胞miRNA表达谱存在巨大差异：差异达2倍以上的miRNA共计34条，其中9条表达上调，25条表达下调。上调最显著（≥4倍）的miRNA为hsa-miR-548m、hsa-miR-498、hsa-miR-1281、hsa-miR-940，共计4条。下调最显著（≥4倍）的miRNA有hsa-miR-98、hsa-miR-31^*^、hsa-miR-16-2^*^、hsa-miR-15b^*^、hsa-let-7e、hsa-miR-26b、hsa-let-7c、hsa-miR-125a-5p、hsa-miR-20b，共计9条。可见在SP中，明显下调的miRNAs基因在数量上超过了在SP中明显上调的miRNA基因。

从图 3基因芯片图谱信号强度对比我们可以看出，SP细胞中miRNA表达丰度要远低于non-SP细胞，这与国外文献^[7]^报道干细胞的miRNA表达水平随着细胞的分化而逐渐增高相符。特别是let-7家族，Shell等^[8]^认为let-7表达水平可以反映肿瘤的分化和恶性程度：let-7表达水平越低表明细胞越原始，癌性越显著。我们通过本次芯片筛查发现在SP细胞中let-7家族成员也显著缺失，这可能与SP细胞高致瘤性、高转移潜力和更高的耐药性相关，需要进一步研究证实。在乳腺癌中，宋尔卫等^[9]^发现let-7家族成员在乳腺癌干细胞中表达下降或缺失最明显；进一步对let-7的功能研究表明，let-7通过抑制*Ras*癌基因和促进*HMGA2*基因表达，负调节乳腺癌干细胞的“干性”与成瘤性。let-7高表达的乳腺癌HMGA2蛋白表达增高，引起肿瘤细胞分化，使肿瘤转移潜能下降；而let-7的缺失使乳腺癌干细胞RAS癌基因高表达，维持了乳腺癌干细胞的“干性”。在肺癌中，let-7可能也发挥了相似的功能，但需要相关研究证实。

在SP中下调的hsa-miR-31^*^、hsa-miR-16-2^*^、hsa-miR-15b^*^、hsa-miR-125a-5p、hsa-miR-29b、hsa-miR-29c均属于抑癌功能miRNA，在它们调控的众多靶基因中，有些是重要的癌基因，例如RhoA是hsa-miR-31靶基因之一^[10]^，具有促进肿瘤转移作用；hsa-miR-15和hsa-miR-16调控*Bcl-2*^[11]^，hsa-miR-29调控*Mcl-1*^[12]^，这两个都是抗凋亡基因；hsa-miR-125靶基因之一是*ErbB2*^[13]^，是一种促进细胞增殖的原癌基因。这些抑癌miRNA在SP中的缺失或低表达会导致对应癌基因高表达，可能在维持肺癌干细胞耐药、抗凋亡、高转移潜力等多种特性中具有重要作用，值得进一步进行研究。

由于本次芯片采用的数据库是目前最新的13.0版本，因此很多具有差异的miRNA在以往均无相关研究报道，特别是在SP中上调的hsa-miR-548m、hsa-miR-498、hsa-miR-1281、hsa-miR-940、hsa-miR-1274b、hsa-miR-1274a等均尚无与肺癌有关的研究，这些上调的miRNA到底在SP细胞中起到何种作用，还需要进一步深入研究。

本研究利用miRNA芯片技术，筛选出肺癌A549细胞株中SP与non-SP细胞差异表达的miRNA，为进一步研究miRNA在肺癌干细胞中的功能及表达调控模式提供了基础。对筛选出的差异miRNA进行验证和功能学的研究将是下一步应开展的工作，通过对miRNA功能及表达调控模式的研究，将对肺癌的预防、诊断和治疗理念提供新的思路和有效途径。
